# Divergent lncRNA *GATA3-AS1* Regulates *GATA3* Transcription in T-Helper 2 Cells

**DOI:** 10.3389/fimmu.2018.02512

**Published:** 2018-10-29

**Authors:** Hunter R. Gibbons, Guzel Shaginurova, Laura C. Kim, Nathaniel Chapman, Charles F. Spurlock, Thomas M. Aune

**Affiliations:** ^1^Department of Pathology, Microbiology, and Immunology, Vanderbilt University, Nashville, TN, United States; ^2^Department of Medicine, Vanderbilt University Medical Center, Nashville, TN, United States; ^3^Program in Cancer Biology, Vanderbilt University, Nashville, TN, United States

**Keywords:** long non-coding RNA, GATA3, GATA3-AS1, R-Loop, lncRNA, epigenetics, transcription, T Cell

## Abstract

Long non-coding RNAs (lncRNAs) possess a diverse array of regulatory functions including activation and silencing of gene transcription, regulation of splicing, and coordinating epigenetic modifications. *GATA3-AS1* is a divergent lncRNA gene neighboring *GATA3*. GATA3 is considered the master regulator of TH2 lineage commitment enabling TH2 effector cells to efficiently transcribe genes encoding cytokines IL-4, IL-5, and IL-13. Here, we show that the *GATA3-AS1* lncRNA is selectively expressed under TH2 polarizing conditions and is necessary for efficient transcription of *GATA3, IL5*, and *IL13* genes, while being sufficient for *GATA3* transcription. *GATA3-AS1* is required for formation of permissive chromatin marks, H3K27 acetylation and H3K4 di/tri-methylation, at the *GATA3-AS1-GATA3* locus. Further, *GATA3-AS1* binds components of the MLL methyltransferase and forms a DNA-RNA hybrid (R-loop) thus tethering the MLL methyltransferase to the gene locus. Our results indicate a novel regulatory function for a divergent lncRNA and provide new insight into the function of lncRNAs in T helper cell differentiation.

## Introduction

Long non-coding RNAs (lncRNAs) represent a new class of regulatory molecules impacting a vast array of biological functions. LncRNAs are defined as >200 nucleotides in length but possess little if any protein-coding potential (1). LncRNA genes are oftentimes named in reference to their neighboring protein-coding genes in the genome (2, 3). Divergent lncRNAs represent one such class and divergent lncRNA gene transcriptional start sites are juxtaposed to their adjacent mRNA gene transcriptional start sites, and may impact transcription of this mRNA by various mechanisms. Previous studies have implied that divergent lncRNAs may have no true function but transcription of the divergent lncRNA may make the gene locus more accessible or alternatively, compete for mRNA gene promoters or proximal enhancers (4, 5). LncRNAs may be localized to the cytoplasm or nucleus. One common mechanism by which lncRNAs act is to recruit histone modifying machinery to gene loci and activate or repress transcription of target mRNA gene loci (6). However, how lncRNAs find their target gene loci, which can be in *cis* or *trans*, is less well understood. One mechanism that has been described is via formation of DNA-RNA hybrids. An example is the lncRNA, *VIM-AS1*, which modifies its neighboring *VIM* gene by forming an R-loop (7). An R-loop can form via G-Rich RNA hybridization to a DNA sequence, forming an RNA:DNA hybrid.

*GATA3-AS1* represents one divergent lncRNA, and shares a promoter region with *GATA3*. The GATA3 transcription factor is considered the master transcriptional regulator of T Helper 2 (TH2) lineage commitment (8) and is required for induction of *IL4, IL5*, and *IL13*, genes required for expression of sentinel TH2 cytokines (9). *GATA3-AS1* levels are elevated in human TH2 cells compared to other T helper cell subsets (10). Expression of *GATA3-AS1* is also increased in reponse to allergen stimulation in patients with allergy or asthma suggesting *GATA3-AS1* may contribute to disease pathogenesis (11).

In this study, we show *GATA3-AS1* is necessary for efficient transcription of *GATA3* as well as *IL5* and *IL13* genes. *GATA3-AS1* both binds to the MLL H3K4 methyltransferase and forms an R-Loop within its own locus to facilitate chromatin remodeling within the *GATA3-GATA3-AS1* locus.

## Materials and methods

### Cell culture, RNA isolation, and quantitative RT-PCR

Human PBMC were cultured under TH0, TH1, TH2, and TH17 polarizing conditions as previously described (10). Cultures were harvested after 5 (TH1, TH2) or 7 days (TH17). Cultures were also re-stimulated with anti-CD3 for 2 days for analysis of effector cells (TH1-E, etc.). Total RNA isolation, cDNA synthesis using poly-A selection and analysis by qPCR was performed as previously described (10). Expression levels of target transcripts were normalized to levels of *GAPDH* using the formula 2^(GAPDHCt−targetgeneCt)^. Primer Pairs used in qPCR reactions are listed in Supplementary Table 1. The study was approved by the institutional review board at Vanderbilt University Medical Center. Written informed consent was obtained at the time of blood sample collection.

### Cell fractionation assay

Human PBMC were incubated to produce TH2 primary and effector populations. Cytoplasmic and nuclear fractions were isolated using a PARIS kit (AM1921, ThermoFisher). RNA from each fraction was isolated as described above.

### RNAi transfections

Human PBMC were incubated for a total of 5 days under TH2-polarizing conditions. Cells were transfected after 2 d of culture with Lipofectamine RNAiMax (Life Technologies) using either an inventoried Silencer Select negative control siRNA or custom designed Silencer Select siRNA for *GATA3-AS1* (DesignID: AD0IWKB and AD1RUQJ), or *GATA3* (DesignID: AD6RNGV amd AD5IPAN) per supplied protocols. Cells were harvested after 5 days and used for either RNA analysis via qPCR, ChIP analysis, ELISA, and Western Blot.

### Enzyme linked immunosorbent assay (ELISA)

Elisa assays were performed according to instructions provided by the kits to analyze IL4 (555194,BD Biosci), IL5 (555202, BD Biosci), IL13 (88-7439-88, Invitrogen), and IFN-γ (555142, BD Biosci) proteins. Cultures were performed as described under RNAi transfections. Cultures were harvested and analyzed by ELISA.

## Western blot

Cells were lysed with RIPA buffer supplemented with protease inhibitors (cOmplete Mini, Roche) and phosphatase inhibitors (PhosStop inhibitor cocktail, Roche). Protein concentration of each sample was determined by Pierce BCA Protein Assay kit. Lysates were subjected to SDS/PAGE followed by blotting with the indicated antibodies. Signal was detected using the IR-dye conjugated secondary antibodies and the Odyssey scanner (Li-cor Biosciences). Antibodies against the following proteins were used: GATA3 (#199428, Abcam) and β-Actin (#47778, Santa Cruz).

### *In-vitro* transcription

Full length *GATA3-AS1* was generated by PCR amplification, agarose gel purified using a QIAquick gel extraction kit (28704, QIAGEN) and cloned into a TOPO-TA dual promoter transcription vector (K462001, ThermoFishter). Clone identify was verified by digestion of plasmids with Spe1 (R0133S, NEB) and Not1 (R0189S, NEB), and DNA sequencing via GENEWIZ. *GATA3-AS1* transcripts were produced via the T7 promoter using the maxiscript T7 transcription kit (AM1312, ThermoFisher). Full length transcripts were transfected into TH0 cells at day 2, at concentrations of 0.5 uM and 0.1 uM similar to RNAi transfections.

### Chromatin immunoprecipitation (ChIP)

ChIP procedures were as previously described (10) using an anti-H3K4me2/3 (ab6000, Abcam), anti-H3K27ac (ab4729, Abcam), or anti-mouse IgG (sc-2025, SantaCruz Biotech). DNA was isolated from beads via phenol chloroform extraction and purified using QiaQuick PCR purification kits. Isolated chromatin was analyzed using SYBR-Green qPCR (Applied Biosystems). Values were expressed as fraction of total input from chromatin samples.

### RNA-immunoprecipitation (RIP)

RIP assays were performed as described previously (10). Briefly, TH2 primary cultures were harvested, lysed, and chromatin sheared by sonication followed by incubation with an isotype IgG control antibody (sc2025, SantaCruz Biotech), anti-WDR5 (ab56919, Abcam), or anti-p300 (ab14984, Abcam) overnight at 4°C. Protein A/G beads (sc2003, SantaCruz Biotech) were added to lysates and incubated at 4°C for an additional 4 h. Beads were pelleted, supernatants harvested, and beads were washed and suspended in Tri-Reagent. RNA was isolated and analyzed via qRT-PCR as described above.

### DNA-RNA hybrid immunoprecipitation (DRIP)

DRIP assays were performed as described (12) using the track 17 in development of protocol. Briefly, TH2 primary cultures were harvested, lysed, and chromatin sonicated at 2 × 10 min (medium, Diagenode Bioruptor) to yield an average chromatin size of ~500 bp. Samples were treated with proteinase K at 65°C overnight. Total nucleic acid was isolated by phenol chloroform extraction. Six micrograms of nucleic acid was rotated overnight with S9.6 antibody followed by incubation with protein A/G beads (SantaCruz Biotech) for 4 h. Beads were subsequently washed 4 times followed by isolation via Tri-Reagent for RNA or phenol chloroform extraction for DNA. Samples were analyzed by qPCR as described above.

## Results

### Selective expression of GATA3-AS1 by TH2 populations

**Figure 1:**
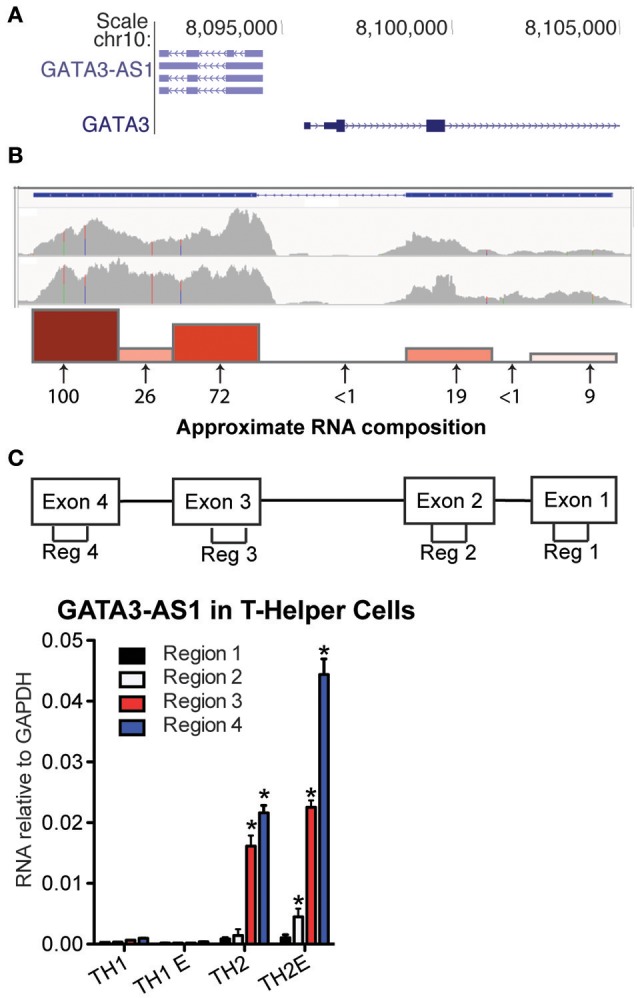
*GATA3-AS1* is expressed in TH2 cells. **(A)** Genomic locations of human *GATA3-AS1* known isoforms and *GATA3* with directions of transcription in sense (>) and antisense orientations (<) from UCSC genome browser (hg19 build). **(B)** Whole genome RNA-seq tracks of *GATA3-AS1* expression in primary (upper) and effector (lower) TH2 cells, Y-axis is in FPKM. Approximate composition of *GATA3-AS1* transcripts in TH2 primary cells was verified by PCR. Arrows indicate approximate genomic locations of seven PCR primer pairs used for the analysis. Numbers below the arrows represent relative transcript amounts at each primer location, and are expressed relative to the highest expressed region. Also depicted as a bar graph above the arrows. **(C)** PCR primer pair locations used to measure exons 1-4 designated as regions 1-4, respectively. Expression levels of *GATA3-AS1* regions 1–4 in TH1, TH1-E, TH2, and TH2-E, subsets. Results are expressed relative to levels of *GAPDH* (*n* = 3), Statistical significance vs. TH1 effector cells was determined by Students *T*-test. *P < 0.05.

Genes encoding *GATA3-AS1* and *GATA3* are adjacent to each other on human chromosome 10 (Figure 1A). *GATA3* is transcribed in the sense orientation and *GATA3-AS1*, the antisense orientation. Transcriptional start sites for *GATA3* and *GATA3-AS1* are separated by ~1200 bp. Thus *GATA3* and *GATA3-AS1* belong to the general class of divergent lncRNA/mRNA pairs. At least six *GATA3-AS1* splice variants have been identified that utilize four major exons. Whole genome RNA-sequencing confirmed multiple regions were transcribed in primary (upper panel) and effector (lower panel) TH2 cultures (Figure 1B). Each exon and intron region of *GATA3-AS1* was evaluated in TH2 primary cells with targeted primer pairs to analyze total expression at these sites. *Gata3-AS1* transcripts contained the four predicted exons, as well as the most downstream intron (Figure 1B). The first and second predicted introns were not present. We decided to examine the four predicted exons, designated regions 1–4, to determine their expression levels in T helper cell cultures. We found that regions 1–4 were selectively expressed in primary and effector TH2 cultures relative to primary and effector TH1 cultures (Figure 1C). Regions 3 and 4 exhibited higher expression levels than regions 1 and 2 consistent with our RNA-sequencing results. We subsequently searched for TH2-specific non-coding RNAs in the mouse genome around *Gata3*. We examined published RNA-seq data (13) and identified three lncRNA genes in the vicinity of Gata3 selectively expressed in TH2 cultures relative to TH1 and TH17 cultures. These were 103 kb, 290 kb, and 336 kb, respectively, 3' of Gata3, named lincR-Gata3-3'S-336K (S = transcribed in sense direction relative to Gata3), lincR-Gata3-3'S-290K, and lincR-Gata3-3'. Given their genomic distances from Gata3, these did not fall into the general divergent lncRNA class.

**Figure 2:**
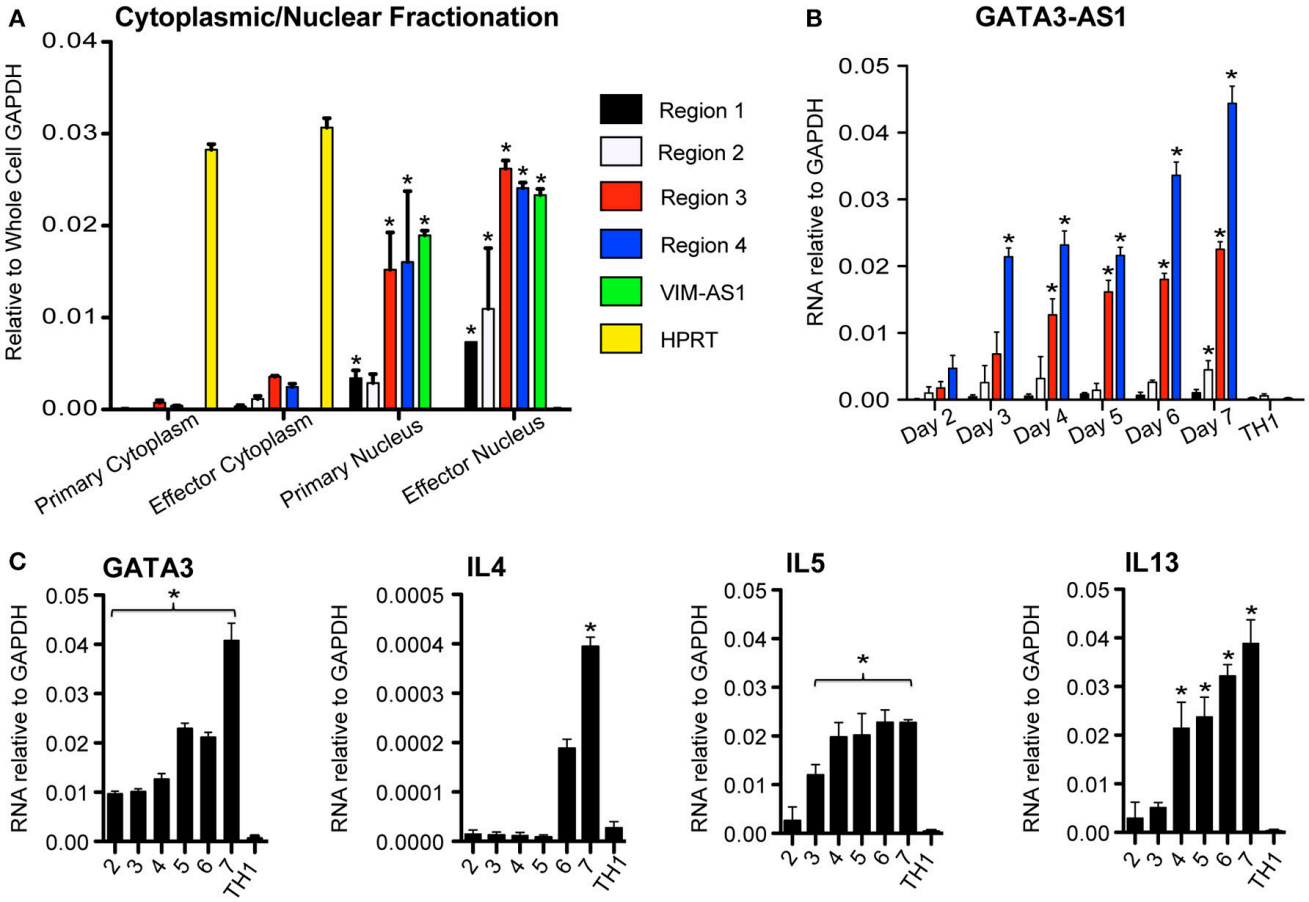
*GATA3-AS1* is localized in the nucleus and expression increases during TH2 cell polarization**. (A)** Cellular and nuclear fractions of primary and effector TH2 populations analyzed via qPCR. Values represent ΔΔCT vs. whole cell *GAPDH. VIM-AS1* lncRNA is a nuclear control, while *HPRT* mRNA represents a cytoplasm specific control. Statistical significance vs. relative cytoplasmic fraction was determined by Students T-test (*n* = 3). *P < 0.05 **(B)** Total PBMCs were cultured under TH1 or TH2 polarizing conditions, and RNA was isolated on consecutive days. Mean ± S.D. gene transcripts of *GATA3-AS1* were quantified via qPCR and normalized to *GAPDH*. **(C)** Similar analyses were completed for *GATA3, IL4, IL5, and IL13*. Statistical significance vs. TH1 effector cells on day 7 was determined by Students *T*-test (*n* = 3). *P < 0.05.

LncRNAs may function in the nucleus or cytoplasm (14). Therefore, we asked if *GATA3-AS1* existed primarily in nuclear or cytoplasmic compartments of TH2 cells. We isolated nuclear and cytoplasmic fractions, isolated total RNA from each fraction and evaluated *GATA3-AS1* levels by PCR. *HPRT* was used as a cytoplasmic specific control mRNA, while *VIM-AS1* represented our nuclear specific control (7). We found that *GATA3-AS1* was located primarily within the nuclear fraction (Figure 2A). We compared the kinetics of induction of *GATA3-AS1* under TH2 polarizing conditions to induction of *GATA3, IL4, IL5*, and *IL13* (Figures 2B,C). We found a continual increase in expression of *GATA3, IL4, IL5*, and *IL13* over time. *GATA3-AS1* displayed a similar increase in transcript levels as a function of time after stimulation. Thus, *GATA3-AS1* induction paralleled induction of genes known to mark the TH2 differentiation pathway.

### Depletion of GATA3-AS1 disrupts induction of GATA3, IL13, and IL5 genes

**Figure 3:**
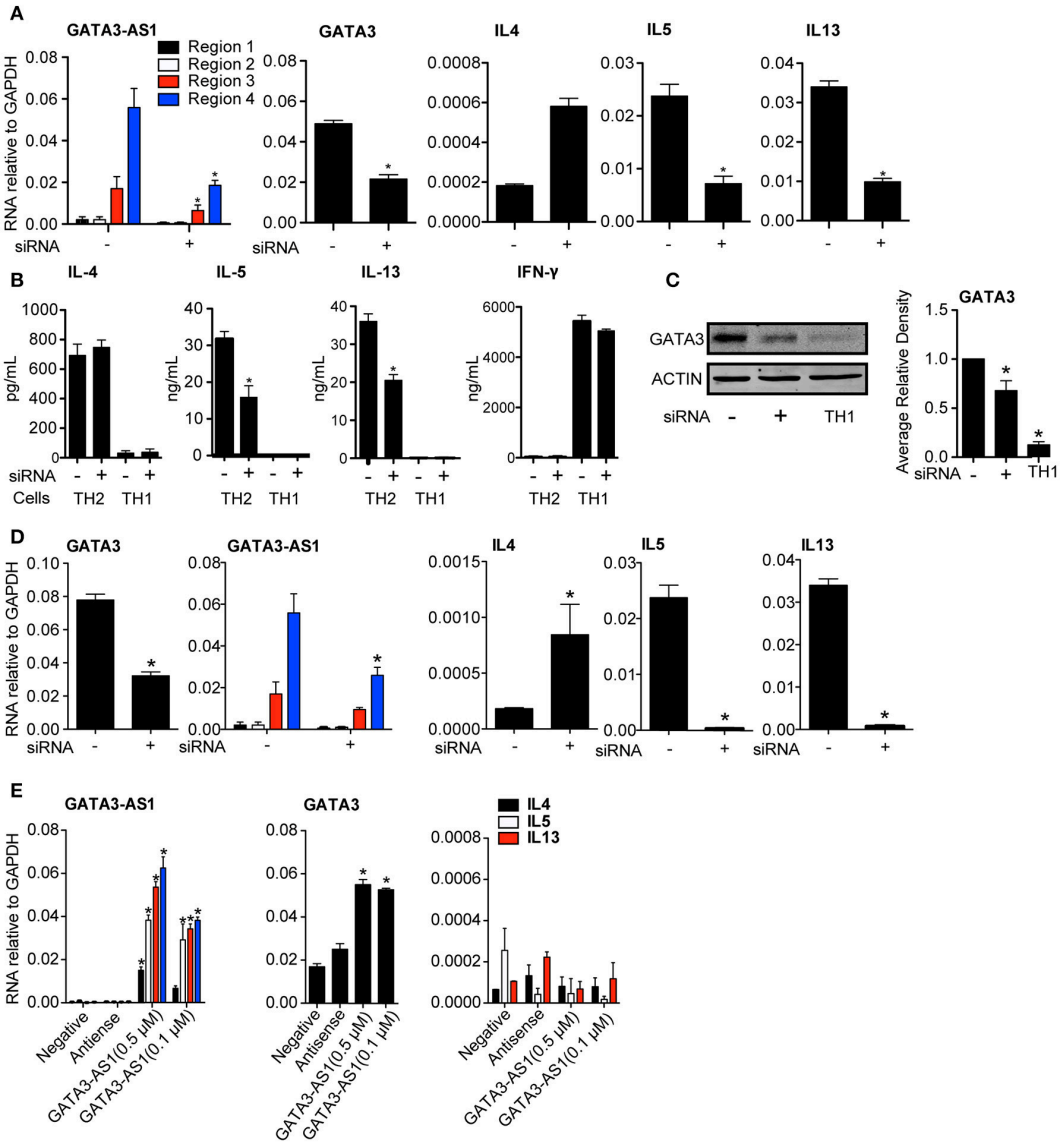
*GATA3-AS1* and *GATA3* form a necessary feed forward loop for TH2 polarization. **(A)** PBMCs were cultured under TH2 polarizing conditions for 2 days and transfected with a *GATA3-AS1* specific siRNA (+) or scrambled siRNA (–). *GATA3-AS1, GATA3, IL4, IL5*, and *IL13* transcripts were determined by qPCR on day 5 and results expressed relative to *GAPDH*. Statistical significance was determined using Students *T*-test by comparing *GATA3-AS1* siRNA knockdown to scrambled control knockdown (*n* = 3). *P < 0.05 **(B)** PBMCs were cultured under TH2 or TH1 polarizing conditions and transfected with *GATA3-AS1* specific siRNA (+) or scrambled siRNA (–). Protein was analyzed by ELISA (for IL-4, IL-5, and IL-13, *n* = 3,) or **(C)** Western Blot (GATA3, Densitometry). Statistics were calculated using a Paired *T*-test compared negative siRNA controls. *P < 0.05. **(D)** Similar to A, but an siRNA specific to *GATA3* was transfected on day 2. Results and statistics similar to A, but (*n* = 4). **(E)** PBMCs were cultured under TH0 conditions for 2 days, and transfected with *GATA3-AS1* transcripts produced from Topo-TA *in vitro* transcription vector. Cells were transfected with a scrambled siRNA (negative), the antisense of *GATA3-AS1*(antisense), *GATA3-AS1* at 500 μM (AS1 High), and *GATA3-AS1* at 100 μM (AS1 Low). Analysis and statistics completed similarly to A and B, (*n* = 5).

To further explore possible functions of *GATA3-AS1*, we designed an siRNA to target the most highly expressed region of the lncRNA, region 4. Total human PBMCs were cultured under TH2 conditions and the *GATA3-AS1* specific siRNA was transfected into cells on day 2. A scrambled siRNA with non-specific target was used as a negative control. Total RNA was isolated on day 5 when cells reached primary stage. We found that *GATA3-AS1* regions 2, 3, and 4 were significantly reduced after transfection compared to transfection with the scambled control siRNA (Figure 3A). Transfection with the *GATA3-AS1* specific siRNA also led to a marked reduction in *GATA3, IL13*, and *IL5* mRNAs. *GATA3-AS1* reduction similarly reduced protein levels of IL5, IL13, (Figure 3B) and GATA3 (Figure 3C). IFN-⋎ production was not significantly impacted by transfection in TH1 cells, showing minimal off target effects. This demonstrates the *GATA3-AS1* transcript is required for effective induction of major TH2 genes. In contrast, *IL4* showed increased transcript levels following the lncRNA knockdown. This is consistent with previous studies showing that knockdown of *GATA3* may induce compensatory effects resulting from increased Stat6 binding to the *IL4* locus (15). Alternatively, more complete depletion of *GATA3* may be required to abrogate *IL4* induction in this model system. Taken together, these results demonstrate that *GATA3-AS1* plays an important role in *GATA3* induction during initial primary TH2 differentiation. Our interpretation is that depletion of *GATA3-AS1* results in loss of induction of *GATA3* under TH2 differentiation conditions leading to reduced *IL13* and *IL5* expression.

Despite expanding literature on lncRNAs, impact of the mRNA partner on transcription of a divergent lncRNA is not well understood. Gata3 induction creates a positive feedback loop, by binding to its own promoter region (16, 17). Because of the shared promoter region, GATA3 protein binding may impact the expression of *GATA3-AS1* during polarization. To test this hypothesis, we decreased *GATA3* levels via siRNA knockdown to evaluate its impact on the expression of *GATA3-AS1* (Figure 3D)*. GATA3-AS1* showed a significant reduction following *GATA3* depletion, indicating *GATA3* is also required for effective *GATA3-AS1* expression. Thus, *GATA3-AS1* is necessary for effective induction of *GATA3*, while *GATA3* subsequently increases *GATA3-AS1* transcript levels creating a kind of feed-forward loop.

Above results demonstrated that siRNA-mediated knockdown of *GATA3-AS1* reduced *GATA3* induction under TH2 polarizing conditions. Therefore, we asked if elevated *GATA3-AS1* was sufficient to induce *GATA3* expression. We used a Topo-TA cloning vector to produce full length transcripts of *GATA3-AS1*, and verified the product via sequencing. We transfected G*ATA3-AS1* into TH0 cells at two different concentrations (0.5 and 0.1 μM), and analyzed *GATA3* and genes encoding TH2 cytokines. We found *GATA3* transcript levels were significantly higher following transfection of *GATA3-AS1*, but this transfection did not induce genes encoding TH2 cytokines (Figure 3E). Thus, *GATA3-AS1* is sufficient to increase *GATA3* in non-polarized T cells, further demonstrating that regulation *GATA3* via *GATA3-AS1* is dependent on the RNA transcript, not on the act of its transcription.

### GATA3-AS1 binds and recruits MLL methyltransferase via WDR5

**Figure 4:**
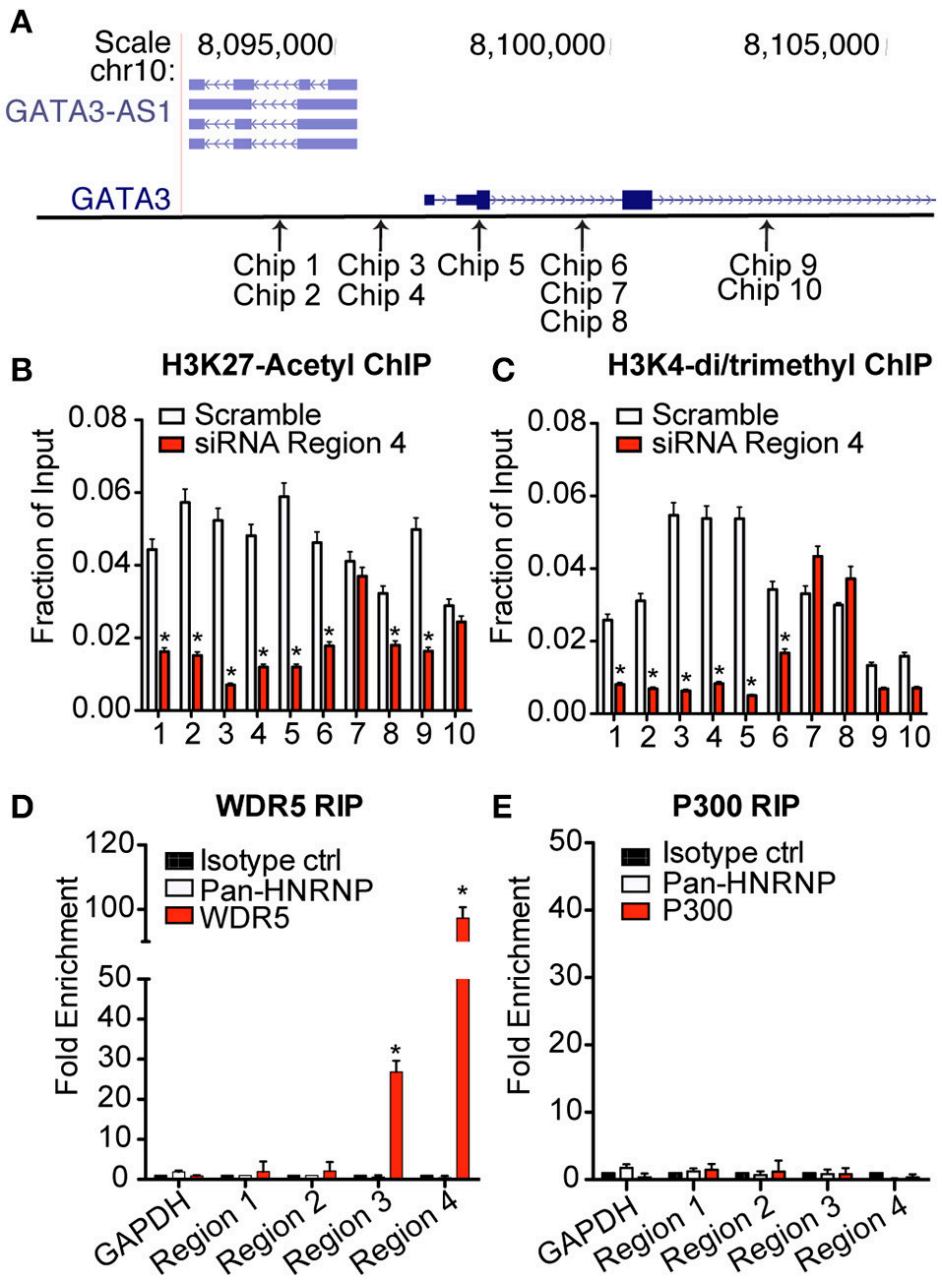
Expression of *GATA3-AS1* changes chromatin marks throughout the *GATA3-AS1*-*GATA3* gene locus. TH2 cultures were transfected with an siRNA specific for *GATA3-AS1* region 4 or a scrambled control siRNA on day 2 as described in Figure 3. Cultures were harvested and processed for ChIP assays on day 5. Results are expressed as fraction of input, mean ± S.D. (*n* = 3). *P < 0.05. **(A)** Approximate genomic positions of PCR primers used for ChIP assays. **(B)** ChIP assays for H3K27ac. **(C)** ChIP asays for H3K4me3. **(D)** WDR5 was immunoprecipitated from TH2 effector whole cell lysates. RNA was isolated from immunoprecipitated WDR5, and *GATA3-AS1* regions 1–4 determined by qPCR. Results are expressed as fold enrichment relative to isotype control, mean ± S.D. (*n* = 3). *P < 0.05. **(E)** As in **(D)**, except an antibody to p300 was employed for RNA immunoprecipitation.

Several lncRNAs localized in the nucleus can modify transcription of target genes by impacting the state of chromatin in the region (18). This can be achieved by facilitating binding of large histone modifying complexes to chromatin and/or direct interaction with DNA (19). To investigate the effect of *GATA3-AS1* on chromatin marks, we performed the knockdown of *GATA3-AS1* as described above and isolated chromatin from TH2 primary cells on day 5. We processed chromatin for ChIP (chromatin immunoprecipitation) assays and performed immunoprecipitations with antibodies specific for either H3K27ac or H3K4me2/3 marks. We designed a series of PCR primer pairs to interrogate genomic regions across *GATA3-AS1* and *GATA3* genes (Figure 4A). Following knockdown of *GATA3-AS1* via siRNA transfection, we found significantly decreased H3K27ac and H3K4me2/3 activating marks across *GATA3-AS1* and *GATA3* genomic regions including the shared promoter (Chip 3–5), *GATA3-AS1* introns and *GATA3* introns (Figures 4B,C). The variation in chromatin marks was not present in chip primers 9 and 10, located after the second exon of the *GATA3* gene. These results indicate that *GATA3-AS1* is required for adequate addition of H3K27ac and H3K42/3me marks toboth its own gene locus, the shared promoter region and the *GATA3* locus.

Previous studies have demonstrated that one mechanism that lncRNAs can alter the epigenetic code is by binding to chromatin modifying complexes to facilitate their recruitment to target genomic loci (20). An example is the MLL H3K4-methyltransferase complex of which WDR5 is an essential component (21). To investigate this possible interaction, we performed RNA immunoprecipitations using antibodies specific for WDR5. We isolated RNA from the immunoprecipitates and analyzed recovery of *GATA3-AS1* transcripts via qPCR. We found that a significantly higher portion of *GATA3-AS1* regions 3 and 4 were immunoprecipitated with antibodies to the WDR5 protein than the pan-hnRNP negative control antibody or an isotype control (Figure 4D). We performed a similar immunoprecipitation using antibodies to p300. p300 is one chromatin modifying enzyme responsible for formation of H3K27ac marks (22). Unlike the MLL methyltransferase, there was no detectable interaction between *GATA3-AS1* and the p300 complex in TH2 cells (Figure 4E). These results demonstrate that *GATA3-AS1* binds the MLL H3K4-methyltransferase complex via interactions involving primarily region 4.

### *GATA3-AS1* forms an R-loop

**Figure 5:**
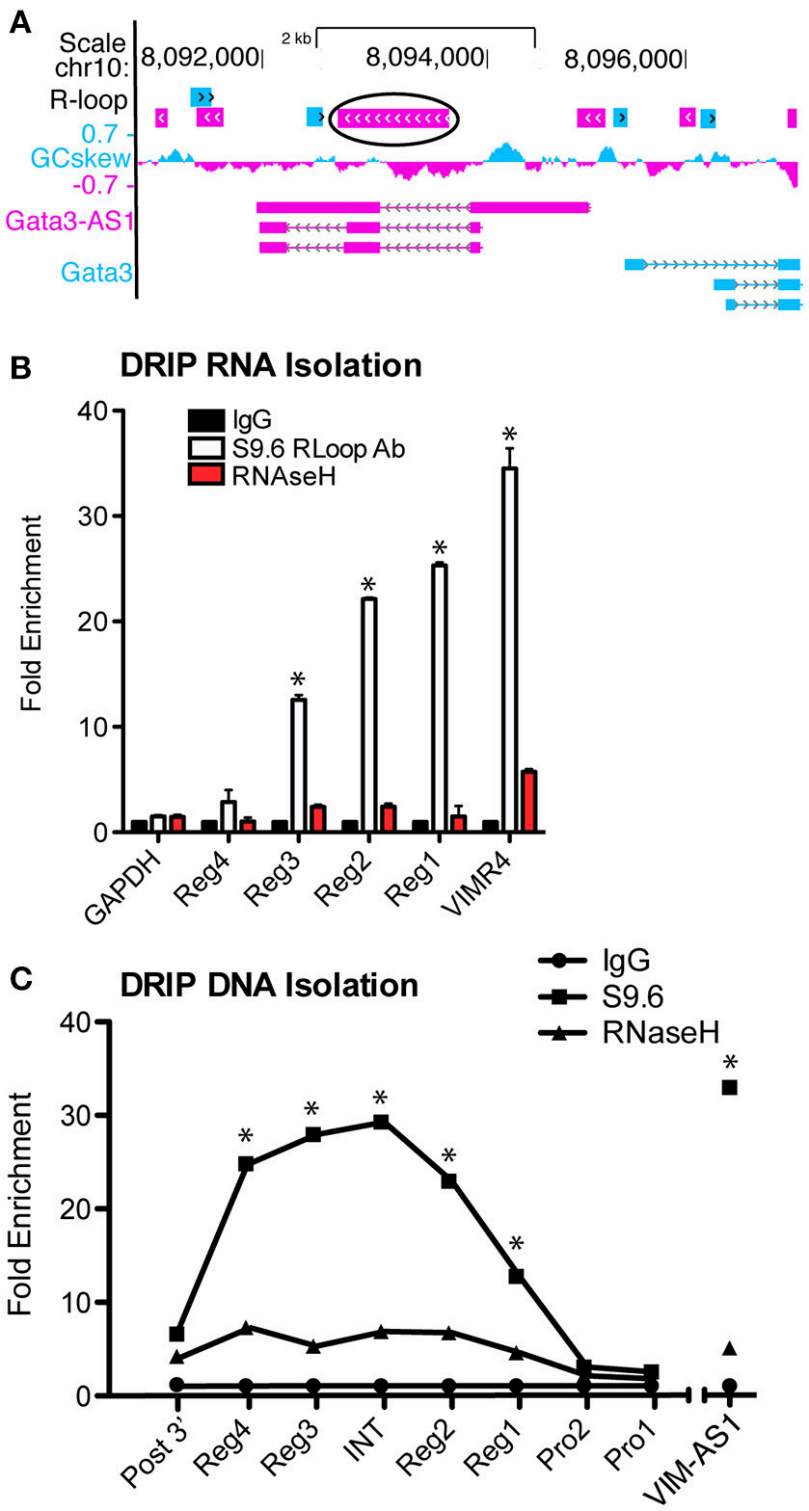
*GATA3-AS1* RNA forms an R-Loop with the central intron of *GATA3-AS1*. **(A)** Predictive R-Loop formations according to the R-Loop database. The largest and most likely R-Loop is circled, located between regions 2 and 3 of *GATA3-AS1*. **(B)** DRIP assay completed with RNA isolation. Results are expressed as fold enrichment relative to the RNase H negative control. Chromatin was treated with RNase to remove R-Loops as an additional control, mean ± S.D. (*n* = 3). *P < 0.05. **(C)** DRIP assay similar to **(B)**, but DNA was isolated from the R-Loop immunoprecipitates. PRO1 and 2 represent the shared promoter region, INT is the central intron of *GATA3-AS1*, and Post3' is past the *GATA3-AS1* transcriptional end site. Statistical evaluations were performed using Students *t*-test vs. Rnase H negative control. All data represent mean± S.D. as fraction of input (*n* = 4). *P < 0.05.

An R-loop is the formation of a DNA:RNA:DNA triplex, in which an RNA binds within its own gene region, instead of being released following polymerase activity as normally occurs. R-loops, when formed at the transcriptional start site may produce an open gene region to promote transcription (23). R-Loops typically form within a region of high G-C skew. In a recent study (7), *VIM-AS1* was found to form an R-loop within the *VIM* gene region. Using the recently developed R-loop database (24), we searched the *GATA3-AS1* and *GATA3* genomic locus and identified the intron between regions 2–3 of *GATA3-AS1* as having a high probability of forming an R-Loop because of the high relative G-C skew throughout the region (Figure 5A). We predicted that formation of an R-Loop may help tether the lncRNA to this region, and to test this hypothesis, we performed a DNA-RNA immunoprecipitation assay (DRIP). The DRIP assay follows a standard ChIP assay protocol, but uses the S9.6 antibody, specific for RNA-DNA hybrid structures (21). The DRIP assay allows for analysis of both the bound RNA transcript, as well as the region of DNA to which it has bound. The RNA typically stays localized to its own gene locus, but the formation of the R-Loop at the transcription start site or the termination site indicates different regulatory functions. We completed the DRIP and isolated RNA samples from immunoprecipitates and found that all four regions of the *GATA3-AS1* lncRNA were enriched in the immunoprecipitates (Figure 5B). Regions 1, 2, and 3 were significantly enriched compared to the *GAPDH* control similar in magnitude to the *VIM-AS1* positive control. We performed a similar immunoprecipitation but isolated the DNA fraction via phenol chloroform extraction. We detected R-Loop formation in an RNase H dependent manner located within the G-C rich central intron of *GATA3-AS1* (Figure 5C). These results, taken together, indicate that *GATA3-AS1* binds the DNA region within the central intron of *GATA3-AS1* and could represent a mechanism by which the divergent lncRNA is able to recruit chromatin modifying complexes to the *GATA3* gene locus.

## Discussion

We show that the lncRNA, *GATA3-AS1*, is selectively expressed under TH2 differentiation conditions, is primarily localized to the nucleus and is necessary for efficient expression of *GATA3* during TH2 lineage commitment and expression of *IL5* and *IL13*, genes encoding cytokines critical for TH2 cell function. Further, *GATA3-AS1* lncRNA is sufficient to induce *GATA3* expression further indicating this divergent lncRNA has biologic function, though it cannot induce total TH2 polarization independent of other factors such as c-Maf or Stat6. *GATA3-AS1* is necessary for chromatin remodeling at the *GATA3-AS1-GATA3* locus. Chromatin modifications are accomplished by the ability of *GATA3-AS1* RNA transcripts to specifically bind the MLL H3K4-methyltransferase complex and tether it to the *GATA3-AS1-GATA3* locus*. GATA3-AS1 also forms* a DNA-RNA hybrid within the *GATA3/GATA3-AS1* locus, termed an R loop suggesting one mechanism by which *GATA3-AS1* may recruit the MLL H3K4-methyltransferase complex to the locus.

In addition to *GATA3-AS1*, lncRNAs are involved in additional aspects of TH cell differentiation and function. For example, the lncRNA, *TH2LCRR*, is selectively transcribed from the TH2 locus control region (LCR) in developing TH2 cells, binds the MLL H3K4-methyltransferase complex, is necessary for chromatin remodeling at *IL4, IL5*, and *IL13* genomic loci and induction of *IL4, IL5*, and *IL13* genes in response to TH2 lineage commitment (10). It is not known if *TH2LCRR* forms a DNA-RNA hybrid as described here but this seems possible. The lncRNA, *LincR-Ccr2-5'AS*, is induced under TH2 polarizing conditions and regulates induction of genes encoding chemokine receptors, including Ccr2, as well as TH2 cell migration, *in vivo* (13). In contrast to *GATA3-AS1* and *TH2LCRR*, its mechanism of action is not known but this lncRNA does not seem to contribute to chromatin remodeling at these gene loci. *IFNG-AS1* (*Tmevpg1, NeST*) is critical for *IFNG* expression during TH1 lineage commitment, by T effector memory cells, and *in vivo* (6, 25–27). The lncRNA, *Rmrp*, plays an important role in TH17 lineage commitment by regulating interactions between *Ror-*γ*t* and its RNA helicase DEAD-box protein 5 (DDX5) binding partner (28). Thus, lncRNAs regulate many facets of TH cell differentiation and function, and *GASTA3-AS1* significantly contributes to TH2 development.

*GATA3-AS1* can be considered one member of the class of divergent lncRNAs in which the transcriptional start site of the lncRNA gene is very close (~100–1000 bp) to the transcriptional start site of the adjacent mRNA gene. In this case, it has not been clear if the lncRNA actually has a function. It has been proposed that these divergent lncRNA/mRNA pairs may share common promoters or proximal enhancers and that competition may impact gene expression rather than the RNA that is produced from the divergent lncRNA gene. Alternatively, the very act of transcription of the lncRNA gene may facilitate chromatin remodeling to alter transcription of the neighboring mRNA gene. Our results are consistent with the interpretation that the *GATA3-AS1* lncRNA, as opposed to these other proposed mechanisms, is necessary for the adequate transcription of *GATA3*. Importantly, the *GATA3-AS1* transcript alone is sufficient to induce an increase in *GATA3* expression. These results are unique among divergent lncRNAs, and show the non-coding RNA molecule as an essential feature of its regulatory capacity. Despite the increase in *GATA3* following *GATA3-AS1* transfection, *GATA3-AS1* transcripts alone could not induce TH2 polarization. Other factors, like STAT6 and c-MAF, also involved in TH2 polarization, may also be necessary.

Formation of RNA-DNA hybrids or R-loops are common, and R-loops are prevalent along chromosomes impacting many cellular processes (21). Similarly, one mechanism by which lncRNAs are thought to act is by recruiting histone modifying enzymes to target gene loci. However, except in a few instances (29), it has not been entirely clear how these lncRNA-enzyme complexes find their target gene loci. The formation of an R-Loop by *GATA3-AS1* may allow the effective recruitment of chromatin modifying enzymes to the *GATA3-AS1*-*GATA3* locus, but further experiments will be necessary to demonstrate this relationship. R-loop formation may represent a general mechanism employed by lncRNAs to find their target gene loci or may be unique to divergent lncRNA/mRNA gene pairs that exist in close proximity in the genome.

## Ethics statement

This study was carried out in accordance with the recommendations of and the protocol was approved by the Institutional Review Board of Vanderbilt University Medical Center. All subjects gave written informed consent in accordance with the Declaration of Helsinki.

## Author contributions

HG and TA designed the overall analytic approach. GS isolated human PBMC. NC helped with analytic approach. CS helped with RNA-seq analysis. HG performed experiments as outlined in the text. LK completed protein based experimetns in Figure 3 and helped prepare written detail for experiments. HG analyzed the data and wrote the mansucript. TA revised the manuscript.

### Conflict of interest statement

The authors declare that the research was conducted in the absence of any commercial or financial relationships that could be construed as a potential conflict of interest.

## Abbreviations

LncRNA: Long non-coding RNA
TH2: T helper 2
ChIP: Chromatin Immunoprecipitation
DRIP: DNA:RNA Immunoprecipitation.

## References

[1] Rinn J Chang H. Genome regulation by long noncoding RNAs. Annu Rev Biochem. (2012) 81:145–66. 10.1146/annurev-biochem-051410-092902 22663078 PMC3858397

[2] Sigova A Mullen A Molinie B Gupta S Orlando D Guenther M. Divergent transcription of long noncoding RNA/mRNA gene pairs in embryonic stem cells. Proc Nat Acad Sci USA. (2013) 110:2876–81. 10.1073/pnas.1221904110 23382218 PMC3581948

[3] Derrien T Guigó R Johnson R The long non-coding rnas: a new (p)layer in the “Dark Matter.” Front Genet. (2012) 2:107 10.3389/fgene.2011.00107 22303401 PMC3266617

[4] Wu X Sharp P. Divergent transcription: a driving force for new gene origination?. Cell (2013) 155:990–6. 10.1016/j.cell.2013.10.048 24267885 PMC3899942

[5] Almada A Wu X Kriz A Burge C Sharp P. Promoter directionality is controlled by U1 snRNP and polyadenylation signals. Nature (2013) 499:360–3. 10.1038/nature12349 23792564 PMC3720719

[6] Gomez J Wapinski O Yang Y Bureau J Gopinath S Monack D. The NeST long ncRNA controls microbial susceptibility and epigenetic activation of the interferon-γ locus. Cell (2013) 152:743–54. 10.1016/j.cell.2013.01.015 23415224 PMC3577098

[7] Boque-Sastre R Soler M Oliveira-Mateos C Portela A Moutinho C Sayols S. Head-to-head antisense transcription and R-loop formation promotes transcriptional activation. Proc Nat Acad Sci USA. (2015) 112:5785–90. 10.1073/pnas.1421197112 25902512 PMC4426458

[8] Zhu J Yamane H Cote-Sierra J Guo L Paul W. GATA-3 promotes Th2 responses through three different mechanisms: induction of Th2 cytokine production, selective growth of Th2 cells and inhibition of Th1 cell-specific factors. Cell Res. (2006) 16:3–10. 10.1038/sj.cr.7310002 16467870

[9] Zheng W Flavell R. The transcription factor GATA-3 is necessary and sufficient for Th2 cytokine gene expression in CD4 T Cells. Cell (1997) 89:587–96. 9160750 10.1016/s0092-8674(00)80240-8

[10] Spurlock CF III Tossberg JT Guo Y Collier SP Crooke PS III Aune TM. Expression and functions of long noncoding RNAs during human T helper cell differentiation. Nat Commun. (2015) 6:6932. 10.1038/ncomms7932 25903499 PMC4410435

[11] Zhang H Nestor C Zhao S Lentini A Bohle B Benson M. Profiling of human CD4+ T-cell subsets identifies the TH2-specific noncoding RNA GATA3-AS1. J Allergy Clin Immunol. (2013) 132:1005–8. 10.1016/j.jaci.2013.05.033 23870669

[12] Halász L Karányi Z Boros-Oláh B Kuik-Rózsa T Sipos É Nagy É. RNA-DNA hybrid (R-loop) immunoprecipitation mapping: an analytical workflow to evaluate inherent biases. Genome Res. (2017) 27:1063–73. 10.1101/gr.219394.116 28341774 PMC5453320

[13] Hu G Tang Q Sharma S Yu F Escobar TM Muljo SA. Expression and regulation of intergenic long noncoding RNAs during T cell development and differentiation. Nat Immunol. (2013) 14:1190. 10.1038/ni.2712 24056746 PMC3805781

[14] Miyagawa R Tano K Mizuno R Nakamura Y Ijiri K Rakwal R. Identification of cis- and trans-acting factors involved in the localization of MALAT-1 noncoding *RNA* to nuclear speckles. RNA (2012) 18:738–51. 10.1261/rna.028639.111 22355166 PMC3312561

[15] Zhu J Min B Hu-Li J Watson C Grinberg A Wang Q Conditional deletion of Gata3 shows its essential function in TH1-TH2 responses. Nat Immunol. (2004) 5:1157–65. 10.1038/ni1128 15475959

[16] Wei G Abraham BJ Yagi R Jothi R Cui K Sharma S. Genome-wide analyses of transcription factor GATA3-mediated gene regulation in distinct T cell types. Immunity (2011) 35:299–311. 10.1016/j.immuni.2011.08.007 21867929 PMC3169184

[17] Scheinman EJ Avni O. Transcriptional regulation of GATA3 in T helper cells by the integrated activities of transcription factors downstream of the interleukin-4 receptor and T cell receptor. J Biol Chem. (2009) 284:3037–48. 10.1074/jbc.M807302200 19056736

[18] Yoon J Abdelmohsen K Gorospe M. Posttranscriptional gene regulation by long noncoding RNA. J Mol Biol. (2013) 425:3723–30. 10.1016/j.jmb.2012.11.024 23178169 PMC3594629

[19] Tsai M Manor O Wan Y Mosammaparast N Wang J Lan F. Long noncoding RNA as modular scaffold of histone modification complexes. Science (2010) 329:689–93. 10.1126/science.1192002 20616235 PMC2967777

[20] Milne T Briggs S Brock H Martin M Gibbs D Allis C. MLL targets SET domain methyltransferase activity to hox gene promoters. Mol Cell (2002) 10:1107–17. 10.1016/S1097-2765(02)00741-4 12453418

[21] Rabello D De Moura C De Andrade R Motoyama A Silva F Altered expression of MLL methyltransferase family genes in breast cancer. Int J Oncol. (2013) 43:653–60. 10.3892/ijo.2013.1981 23754336

[22] Ito T Ikehara T Nakagawa T Kraus WL Muramatsu M. p300-mediated acetylation facilitates the transfer of histone H2A–H2B dimers from nucleosomes to a histone chaperone. Genes Dev. (2000) 14:1899–907. 10.1101/gad.14.15.1899 10921904 PMC316828

[23] Ginno P Lott P Christensen H Korf I Chédin F. R-Loop formation is a distinctive characteristic of unmethylated human CpG island promoters. Mol Cell (2012) 45:814–25. 10.1016/j.molcel.2012.01.017 22387027 PMC3319272

[24] Jenjaroenpun P Wongsurawat T Sutheeworapong S Kuznetsov V. R-loopDB: a database for R-loop forming sequences (RLFS) and R-loops. Nucleic Acids Res. (2016) 45:D119–27. 10.1093/nar/gkw1054 27899586 PMC5210542

[25] Collier S Collins P Williams C Boothby M Aune T. Cutting edge: influence of tmevpg1, a long intergenic noncoding RNA, on the expression of Ifng by Th1 Cells. J Immunol. (2012) 189:2084–8. 10.4049/jimmunol.1200774 22851706 PMC3424368

[26] Collier SP Henderson MA Tossberg JT Aune TM. Regulation of the Th1 genomic locus from Ifng through Tmevpg1 by T-bet. J Immunol. (2014) 193:3959–65. 10.4049/jimmunol.1401099 25225667 PMC4185266

[27] Collins PL Henderson MA Aune TM. Diverse functions of distal regulatory elements at the IFNG locus. J Immunol. (2012) 188:1726–33. 10.4049/jimmunol.1102879 22246629 PMC3273639

[28] Huang W Thomas B Flynn R Gavzy S Wu L Kim S. DDX5 and its associated lncRNA Rmrp modulate TH17 cell effector functions. Nature (2015) 528:517–22. 10.1038/nature16193 26675721 PMC4762670

[29] Sigova A Abraham B Ji X Molinie B Hannett N Guo Y. Transcription factor trapping by RNA in gene regulatory elements. Science (2015) 350:978–81. 10.1126/science.aad3346 26516199 PMC4720525

