# Supplementary material for: Divergent lncRNA GATA3-AS1 Regulates GATA3 Transcription in T-Helper 2 Cells
Source: Front Immunol. 2018 Oct 29;9:2512. doi: 10.3389/fimmu.2018.02512 (PMC6215836; doi:10.3389/fimmu.2018.02512)
Supplement: Supplementary file 1 [file Table_1.DOCX]

Supplementary Material

Divergent lncRNA *GATA3-AS1* regulates *GATA3* transcription via R-loop formation^1^

**Hunter R. Gibbons*, Guzel Shaginurova^†^, Nathaniel Chapman^†^, Charles F. Spurlock, III^†^, and Thomas M. Aune*^,†,2^**

*** Correspondence:** Thomas M. Aune, e-mail: tom.aune@vanderbilt.edu

**Supplemental Table 1**

**Supplementary Table 1.** Primers used in Quantitative PCR Reactions

| Primer Name | Forward | Reverse |
| --- | --- | --- |
| Region 1 | TTTGAGCCACTGCATCCCCTAG | CCCTGATTTCAAACAAACATCA |
| Region 2 | GAAAAATGCTTCTCCGCGCGTC | CCTCAGAAGTCAGCATTTTCAAG |
| Region 3 | CCTCGGCCTGGTCTAGCGAGTC | CTTTTTCCCAAGAGGACACACA |
| Region 4 | CAGAAGAAAGAAGCGGAGCCGAGCCGA | GCCGCTTCACACCGTTTTTATT |
| GATA3 | GGGCAGATGACAGGTTCCAA | CCAATGCATACCAAGGGGGT |
| IL4 | TCTCTGTCCGGTTGGAGGTT | GCGAGTGTCCTTCTCATGGT |
| IL5 | CTCTTGGAGCTGCCTACGTG | TTTCCACAGTACCCCCTTGC |
| IL13 | CCTGATCAACGTGTCAGGCT | TGAACTGTCCCTCGCGAAAA |
| GAPDH | AGCCACATCGCTCAGACAC | GCCCAATACGACCAAATCC |
| ChIP #1 | GGGCGCCTATCCGCTAATTT | TTCTAAAGGTGGGGGTTGCC |
| ChIP #2 | GTCTGGGTGGTTTGAGGCTG | GTGACGTCAAACCCAGTGTG |
| ChIP #3 | TTGACTGTGGGAGAAACGCC | TCATTCCCCGAGTACGCTCA |
| ChIP #4 | GCTCGGGAAAGAGGTGACAA | ATGCAGGGTGTTTGGGAGAG |
| ChIP #5 | TCGCAGAATTGCAGAGTCGT | TGGGAAGCAAAGGTGAGCAA |
| ChIP #6 | TGCATTGGGGAAGCAGAGTT | GCTAGGCAGCGTTTGCATTT |
| ChIP #7 | AGGAGGAGAGGAGGCCATTT | AGGTGTGCTGTTCTCGGTTT |
| ChIP #8 | CCCCAATGAGCTGGGATAGG | CTCGGCCTTCCATTTGGTCT |
| ChIP #9 | CGAACTGTCAGACCACCACA | CGAGCCCTGTTCTTGCTGAT |
| ChIP #10 | GGACTGGGATCAGCAAGAACA | GCATACCAAGGGGGTTGTGT |
| INT | CGATCAACCTGGACGCCT | GTGACGTCAAACCCAGTGTG |
| Post3’ | CCCCAGTCGCCCTTTACAAT | ACAATAACCGCCCAGAGACG |
| PRO1 | TTGACTGTGGGAGAAACGCC | TCATTCCCCGAGTACGCTCA |
| PRO2 | GCTCGGGAAAGAGGTGACAA | ATGCAGGGTGTTTGGGAGAG |
| VIM-AS1 | ACCGGACCCCTCTGGTTC | ACCCTGGGGTGCTGAAAA |
| HPRT | CCTGGCGTCGTGATTAGTGA | CGAGCAAGACGTTCAGTCCT |
